# Three rare etiologies of urinary retention in pediatrics: A case series and review of the literature

**DOI:** 10.1002/ccr3.8125

**Published:** 2023-11-02

**Authors:** Pooya Hekmati, Hamid Arshadi, Hooman Kamran, Abdol‐Mohammad Kajbafzadeh, Mazyar Zahir, Hossein Amirzargar, Seyyed Mohammad Ghahestani

**Affiliations:** ^1^ Department of Pediatric Urology, Pediatric Urology and Regenerative Medicine Research Center, Pediatrics Center of Excellence Tehran University of Medical Sciences Tehran Iran; ^2^ Student Research Committee Shiraz University of Medical Sciences Shiraz Iran; ^3^ Urology and Nephrology Research Center Shahid Beheshti University of Medical Sciences Tehran Iran

**Keywords:** bladder diverticula, mullerian duct cyst, pediatrics, ureteral stump syndrome, urinary retention

## Abstract

Etiology of urinary retention in pediatric age differs significantly from adults and the elderly. Therefore, a comprehensive diagnosis is crucial before specific treatment. Every effort must be made to minimize invasive procedures as far as possible in children.

## Graphical abstract

The etiology.

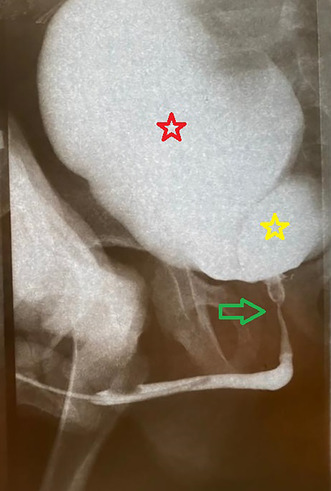

## 1 INTRODUCTION

Urinary retention (UR) is the inability to pass urine in a person with a fully distended urinary bladder.^1^ While benign prostatic hyperplasia, the primary cause of acute urinary retention (AUR) in an adult male is well known; the etiologies in the pediatric group are less investigated^1^, ^2^ and only a few studies have been conducted on UR in children.^2^, ^3^, ^4^

Generally, UR can be divided into acute and chronic forms. In contrast to chronic UR, which mostly presents painlessly, AUR is often a painful condition.^5^ Although there are several classification systems, pediatric UR is often classified according to the underlying etiology. Mechanical, inflammatory, infectious, neurologic and behavioral etiologies have been reported in the literature.^3^ Previous studies have suggested mechanical etiologies as one of the most prevalent causes of pediatric AUR.^2^, ^3^

In this study, we presented three uncommon and complex mechanical types of AUR in children. These included a bladder diverticulum, a Müllerian duct cyst (MDC), and a ureteral stump syndrome. We then reviewed the literature, described existing treatment modalities and delineated our management strategy. This manuscript was prepared following the CARE guidelines (https://www.carestatement.org).

## 2 CASES PRESENTATION

### 2.1 Bladder diverticulum

**FIGURE 1:**
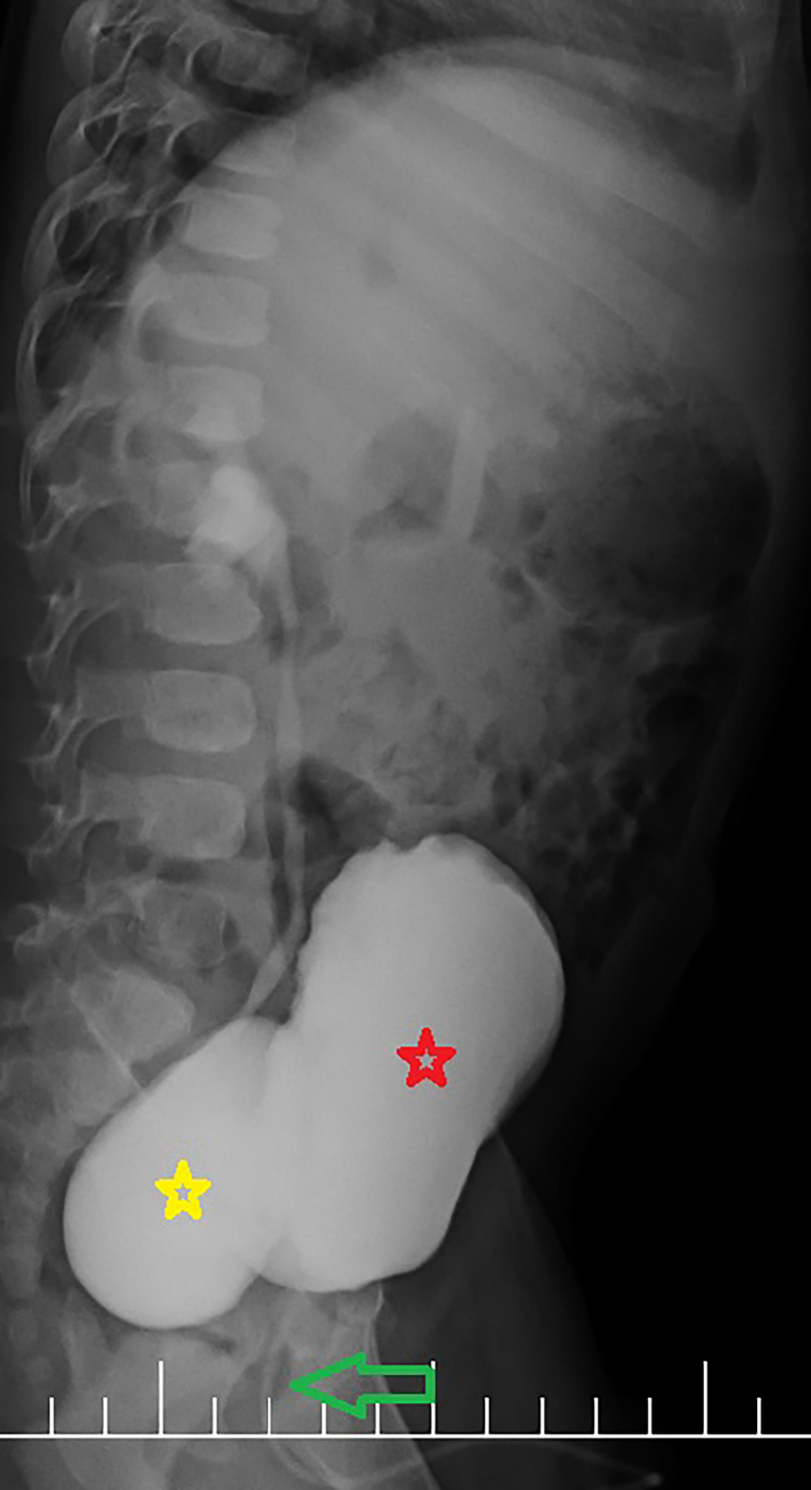
A large bladder diverticulum with ipsilateral vesicoureteral reflux (Grade 3) which compressed the bladder neck and posterior urethra during voiding. A narrow urethral course due to weak and poor contrast flow is notable; red star: urinary bladder, yellow star: bladder diverticulum, green arrow: urethra.

The patient was a 14‐month‐old male infant with AUR. Upon further workup, we found that the boy had been experiencing voiding difficulties since birth. Abdominopelvic ultrasonography (US), voiding cystourethrography (VCUG), and urodynamic studies (UDSs) were performed for the patient. Abdominopelvic US and VCUG studies revealed remarkable compression caused by a bladder diverticulum at the level of the bladder neck and posterior urethra, hampering successful voiding despite bladder fullness and the patient straining to void. Additionally, an ipsilateral vesicoureteral reflux (VUR) was observed (Figure 1).

**FIGURE 2:**
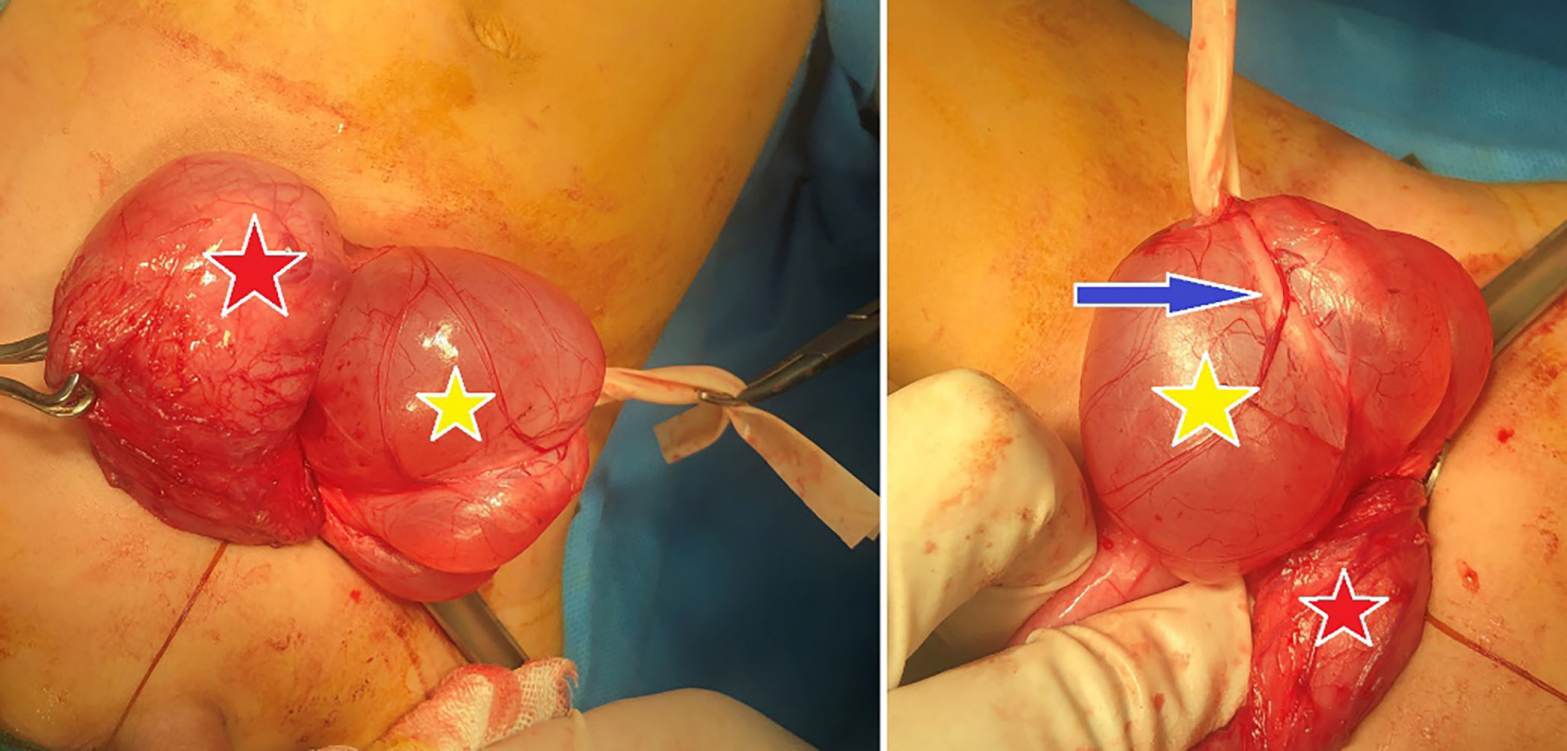
Intraoperative images of bladder diverticulum resection; red star: urinary bladder, yellow star: bladder diverticulum, blue arrow: ureter.

Open surgical resection, utilizing intra‐ and extravesical approaches, was performed for him (Figure 2). The ureter was then reimplanted. The operation was uneventful, and the patient voided freely after postsurgical foley catheter removal. We followed the patient with a UDS 6 months after surgery. The UDS was mostly normal (i.e., normal bladder compliance, no sign of bladder over activity, PdetQmax = 64 cmH_2_O, Qmax = 21 mL/s) except for some degree of dysfunctional voiding. Follow‐up abdominopelvic USs were also performed every 6 months, and he had four normal abdominopelvic USs after the operation. Findings from the last abdominopelvic US were as follows: no sign of hydroureteronephrosis, bladder wall thickness = 4.5 mm, post‐void residue (PVR) = 10 mL. A uroflowmetry was also performed on the latest follow‐up which was satisfactory (i.e., Qmax = 23 mL/s).

### 2.2 Müllerian duct cyst

**FIGURE 3:**
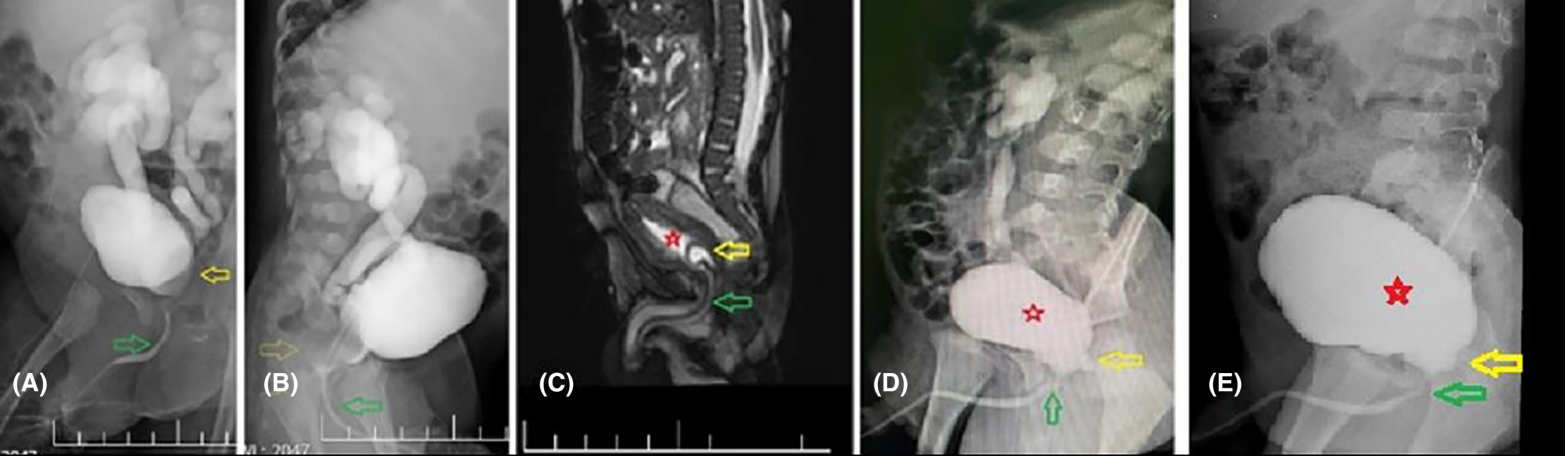
(A, B) Huge obstructing cystic lesion at the level of the bladder neck with bilateral high‐grade vesicoureteral reflux (VUR). (C) Magnetic resonance (MR) urography showing cystic lesion arising from the posterior urethra, compressing the posterior urethra and bladder neck. The lesion did not contain any solid components. (D) First follow‐up voiding cystourethrography (VCUG) showed that the grade of VUR on both sides decreased remarkably, with acceptable contrast flow in the urethra and outpouching of the nonobstructive/empty Müllerian duct cyst (MDC) remnant at the level of the bladder neck and posterior urethra. (E) Second follow‐up VCUG performed 3 years postoperatively. Red star: urinary bladder, yellow arrow: MDC, green arrow: urethra.

The patient was a 3‐day‐old boy with AUR who was unable to void and had dribbling since birth. When the urethral catheter insertion failed, a cystostomy tube was inserted for him. The patient then underwent an investigational cystoscopy, where a large obstructing mass in the posterior urethra and bladder neck with severe bladder trabeculation was observed. An internal foley catheter was inserted during cystoscopy, and the patient underwent further workup (Table 1). Findings of our initial VCUG (Figure 3A,B) and initial magnetic resonance urography (Figure 3C) confirmed the presence of a large obstructing cystic lesion without any solid components at the level of the bladder neck.

**TABLE 1:** Timeline of the patients' clinical courses.

Patient	Diagnostic or therapeutic procedure	Date
One	Admission in emergency department due to urinary retention	12/28/2018
	Initial abdominal ultrasound	12/29/2018
	Initial urodynamic study	12/29/2018
	Initial voiding cystourethrography	12/31/2018
	Initial cystoscopy	01/02/2019
	Open surgical resection of the bladder diverticulum	04/13/2019
	Postsurgical follow‐up visit (urodynamic study and abdominal ultrasound)	10/09/2019
	Latest follow‐up visit (uroflowmetry)	05/07/2023
Two	Admission in emergency department due to urinary retention	07/12/2019
	Insertion of cystostomy tube	07/13/2019
	Cystoscopy and insertion of internal Foley catheter	07/23/2019
	Initial voiding cystourethrography	07/26/2019
	Initial magnetic resonance urography	07/28/2019
	Second cystoscopy and initial transurethral cyst puncture and aspiration	07/30/2019
	Post‐interventional follow‐up abdominal ultrasound	10/28/2019
	Third cystoscopy and secondary transurethral cyst puncture and aspiration	06/07/2020
	First follow‐up voiding cystourethrography	06/20/2021
	Follow‐up dimercaptosuccinic acid (DMSA) renal scan	01/15/2022
	Second follow‐up voiding cystourethrography	05/22/2022
	Latest follow‐up visit (abdominal ultrasound)	11/29/2022
Three	Initial admission due to urinary tract infection	11/17/2017
	Second time admission due to pyonephrosis and sepsis	07/28/2018
	Initial voiding cystourethrography	08/10/2018
	Initial cystourethroscopy	01/10/2019
	Dimercaptosuccinic acid (DMSA) renal scan	07/02/2019
	Left nephrectomy with partial ureterectomy due to severe renal scarring	08/02/2019
	Weak urine stream and recurrent episodes of urinary retention	–
	Abdominopelvic ultrasound and voiding cystourethrography	09/03/2019
	Ureteral stump resection	09/06/2019
	Postsurgical follow‐up visit	11/06/2019
	Latest follow‐up visit	12/03/2022

Based on the patient's age and acute symptoms, we performed transurethral cyst aspiration after completing his workup in the second week of life. The neonate could void after the first procedure but in an interrupted pattern. Therefore, we started clean intermittent catheterization (CIC) and reevaluated the patient at the age of 3 months. The baby had no episodes of urinary tract infection (UTI) 3 months after the initial transurethral cyst puncture. Abdominopelvic US also showed remarkable improvement in hydronephrosis on both sides. We performed a second‐look cystoscopy at the age of 11 months. The cyst remnant was visible at the prostatic urethra, but its volume decreased remarkably. We performed aspiration and multiple punctures at the dependent part of the cyst in the prostatic urethra. The aspirated fluid was light yellow, and cytological evaluation revealed no malignant cells or any types of germ cells. The baby was CIC‐free after the second procedure.

One year after the second aspiration procedure, the first follow‐up VCUG was obtained which showed a notable improvement in VUR grade on both sides. Initially, the VUR grade was 5 on both sides before the initial cyst puncture and aspiration (Figure 3A,B) but improved to Grade 3 on the right side and Grade 2 on the left side after the two consecutive procedures (Figure 3D). A dimercaptosuccinic acid (DMSA) renal scan was also obtained which did not demonstrate any new renal scar. The patient's VUR completely resolved by age 3 as evaluated by a second follow‐up VCUG (Figure 3E). In the last follow‐up visit, the child had an acceptable urine stream and a PVR of less than 5 mL was reported in the abdominal US. The patient has not had any urinary retention episodes and has been UTI‐free after the second aspiration procedure.

### 2.3 Ureteral stump syndrome

A 3‐year‐old boy with a right‐sided single functional kidney and hydronephrotic kidney with an obstructing–refluxing megaureter on the left side has been under routine follow‐up due to antenatal hydronephrosis and voiding dysfunction. The patient received prophylactic antibiotic therapy due to Grade 5 VUR on the left side and poor functioning hydronephrotic kidney (less than 10%) until the age of 2. The boy suffered repetitive episodes of UTI when he started toilet training at age 3. His UTIs could not be controlled with prophylactic antibiotics, changing his voiding behaviors, or even by performing a complete course of biofeedback therapy. The patient also had high PVR due to high‐grade VUR. At this stage, a cystourethroscopy was planned to better evaluate the abnormally high PVR and assess for possible urethral pathology. The left ectopic ureter was located at the posterior urethra just below the bladder neck with a relatively normal‐appearing opening. A new DMSA renal scan revealed a hydronephrotic left kidney with less than 5% activity. Therefore, we planned a nephrectomy with a partial ureterectomy.^6^, ^7^

**FIGURE 4:**
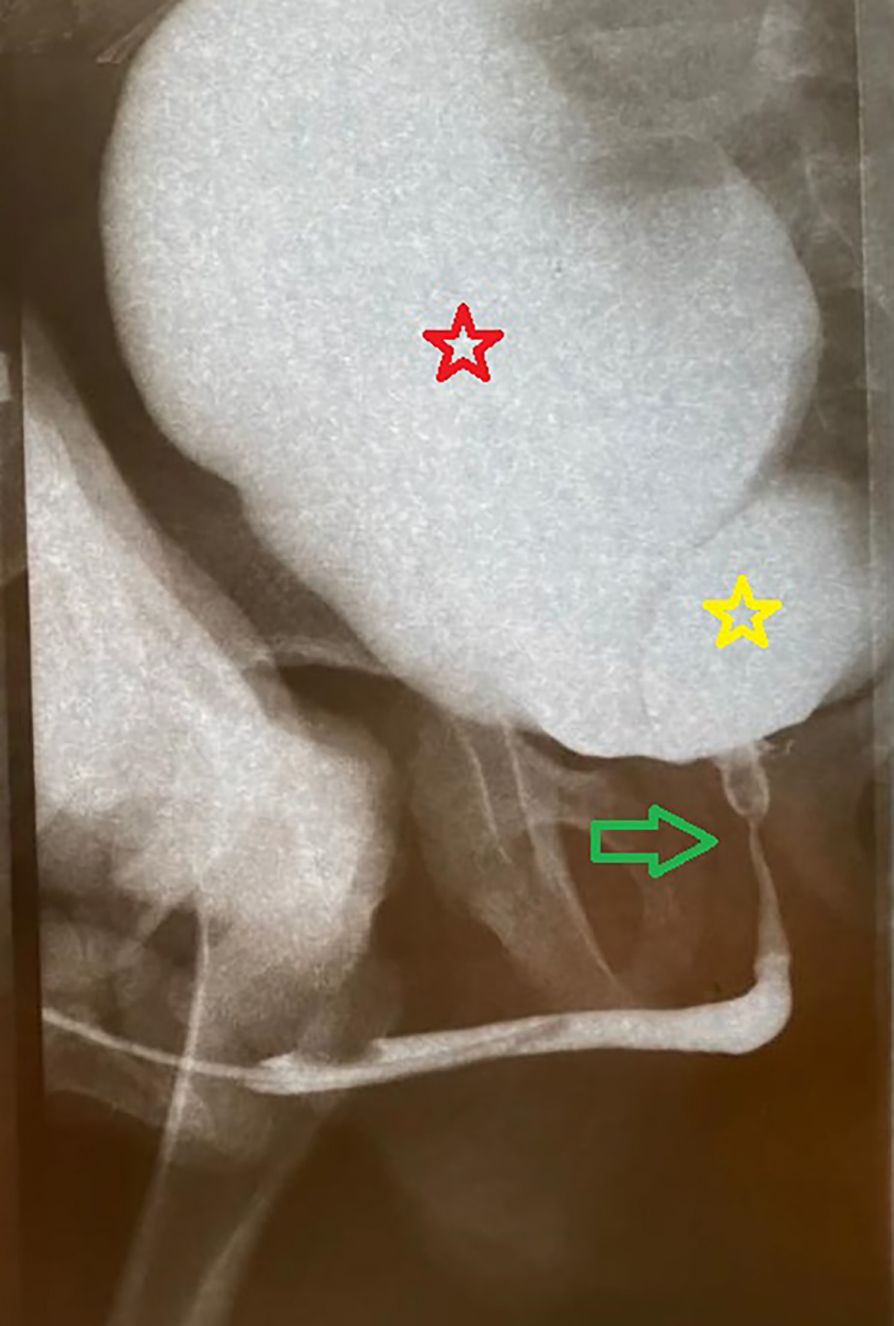
Large refluxing‐obstructing ureteral stump compressed bladder neck and posterior urethra. After catheter removal, the patient could not accomplish a voiding cliché. Therefore, urethral visualization/evaluation was performed using a retrograde approach. Red star: urinary bladder, yellow star: ureteral stump, green arrow: urethra.

We did not perform an extensive dissection beneath the bladder neck, trigone, and posterior urethra to remove the ureter completely; as an extensive dissection of these areas could predispose the patient to further voiding dysfunction. After the surgery, the patient experienced a decrease in his urine stream compared to his preoperative condition and developed repeated episodes of UR. Consequently, our patient was started on CIC therapy. Abdominopelvic US and VCUG were performed. VCUG revealed a refluxing dilated ureteral stump, with a ureteral orifice located in the posterior urethra, which caused narrowing of the posterior urethra and compressed the bladder neck during micturition (Figure 4).

Surgical removal of the ureteral stump was planned. The distal part of the stump was dissected from the surrounding tissues. The ureteral stump was then opened; utilizing a longitudinal incision on its ventral aspect down to the bladder neck level. Thus, we accessed the ureteral orifice using an intraluminal approach. Lastly, at the level of ureteral insertion into the posterior urethra, we oversewn the orifice with an absorbable suture and excised only the mucosal layer of the distal part of the ureteral stump. This method avoids extra dissection of the area beneath the trigone, the bladder neck, and the posterior urethra. The patient was symptom‐free and had no other UR incidences during the 5‐year follow‐up. Table 1 provides a succinct timeline of the patients' evaluation, therapeutic management, and postoperative follow‐up.

## 3 DISCUSSION

UR is relatively rare in pediatric age. A wide variety of etiologies have been attributed to causing AUR in children. For instance, in a study by Nevo et al.^3^ on 56 children with UR, the authors reported that mechanical obstruction (25%) was the most common cause of UR, followed by infectious/inflammatory causes (18%), fecal impaction (13%), neurologic diseases (11%), gynecologic pathologies (7%), and behavioral etiologies (5%). In a more recent and larger study by Schmidt et al.^2^ involving 97 pediatric patients with AUR, infection and inflammation (41.2%), functional disorders (19.6%), mechanical obstruction (12.4%), trauma (11.3%), and neurogenic disorders (3.1%) were identified as the causes of AUR. This wide variety of etiologies and their complex nature, usually calls for a comprehensive diagnostic approach including VCUG, Abdominopelvic US, computed tomography (CT), magnetic resonance imaging (MRI), and occasionally, endoscopic investigation, to identify the underlying pathology.

In the present study, we presented three rare cases of mechanical UR in children. We discussed their etiologies with the subsequent managements and reviewed the previous evidence in each case. In order to conduct a thorough literature review, the previous studies on UR caused by bladder diverticulum, MDC, and ureteral stump syndrome in children were found by searching the following keywords in PubMed (March 2023): (“Urinary Retention”[MeSH Terms] OR “retention*”[Title/Abstract]) AND (“diverticul*”[Title/Abstract] OR “mullerian*”[Title/Abstract] OR “stump*”[Title/Abstract]). Additional articles were added from the bibliography of the retrieved manuscripts. Only English articles were included. Studies which included patients above 18 years of age, reported UR due to other etiologies, or their full text was not accessible, were excluded. With regards to bladder diverticula causing UR, 20 articles, including 49 cases, were found, while only four articles, including five cases, were found for MDC.^8^, ^9^, ^10^, ^11^, ^12^, ^13^, ^14^, ^15^, ^16^, ^17^, ^18^, ^19^, ^20^, ^21^, ^22^, ^23^, ^24^, ^25^, ^26^, ^27^, ^28^, ^29^, ^30^, ^31^ Besides, no report for UR due to ureteral stump syndrome was found with the mentioned keywords.

### 3.1 Bladder diverticulum

According to the literature, at least four different types of bladder diverticula (i.e., congenital, acquired, iatrogenic, and syndromic) can be identified in pediatric age group. Congenital bladder diverticula are mucosal protrusions through the weak points of detrusor muscle. They are relatively rare in children (0.7%–1.7%) and usually occur in men.^16^, ^32^ Patients with congenital bladder diverticula are usually asymptomatic, although they may present with UTI, stone formation, VUR, hematuria, UR, and in rare cases, diverticular rupture or ureteral obstruction.^13^, ^16^, ^32^, ^33^, ^34^, ^35^, ^36^ A low‐lying congenital bladder diverticulum may cause bladder outlet obstruction; by impinging on the bladder neck and urethra after being filled with urine.^37^ Acquired bladder diverticula occur due to an aberrant increase in intravesical pressure. Anatomical or functional obstruction is the primary cause of this type of obstruction. These patients usually have simultaneous voiding symptoms, such as urgency, frequency, hesitancy, and abdominal voiding. Iatrogenic diverticula are rare and usually occur following ureteral reimplantation, cystostomy tube insertion, rectovesical fistula repair, and other surgical interventions on the urinary bladder.^38^ The last type of bladder diverticula exist in syndromic conditions, such as prune belly syndrome, Williams syndrome, and Ehlers–Danlos syndrome.^13^

Most studies concur on the efficacy of VCUG as a suitable diagnostic method in these patients. Voiding plains must be obtained, as in some cases, the bladder diverticulum can only be detected on voiding images. Oblique images are also advised for better anatomical demonstrations.^28^ Additionally, Garat et al.^34^ suggested that pre‐ and postoperative UDSs can be obtained in primary (nonobstructive) congenital bladder diverticula to better evaluate the associated functional or anatomical abnormalities.

Congenital bladder diverticulum treatment must be individualized. Asymptomatic children, especially those without ipsilateral VUR, can be kept under observance. Intervention should be reserved for children who exhibit symptoms related to the bladder diverticulum. Expectant management is also preferable in patients with congenital connective tissue abnormalities, such as Menkes' or Ehlers–Danlos syndromes. Considering the remarkable risk of hemorrhage, poor wound healing, and higher rates of bladder diverticulum recurrence; it is better to follow them unless they present with symptoms, such as repeated UTI and outlet obstruction.^21^, ^39^, ^40^, ^41^

Surgical intervention should be considered in symptomatic bladder diverticula patients. Although transurethral procedures are not commonly employed,^37^ some studies have reported notable outcomes following endoscopic fulguration of bladder diverticula in adult patients.^42^, ^43^ Surgical excision of the bladder diverticulum by open or laparoscopic/robotic approaches is the recommended treatment for symptomatic patients. The transvesical approach allows complete resection of the bladder diverticulum and possible ureteral reimplantation. In huge bladder diverticula, a combination of extra‐ and intravesical approaches may also be used.^12^, ^33^, ^34^ Laparoscopic surgery,^44^, ^45^ pneumovesicoscopic surgery,^46^ and robotic‐assisted diverticulectomy^47^ are all acceptable modalities for bladder diverticulum excision. Ureteral reimplantation has not been advised for all patients except those with ipsilateral VUR. Cutaneous vesicostomy is the primary treatment for symptomatic neonates or very young children, and diverticulectomy is postponed until the first year of life.^12^ In these patients, the physician should carefully observe the postoperative intravesical pressure because removal of the diverticulum and the subsequent reduction in the overall bladder capacity can significantly increase it.

### 3.2 Müllerian duct cyst

Müllerian duct anomalies result from abnormal development and fusion of the Müllerian ducts in females and failure of complete regression in males. Sertoli cells excrete the Müllerian inhibiting substance (MIS), which causes Müllerian duct regression in the male fetus. Abnormalities in MIS production, timing of its production, and/or its receptors may lead to Müllerian duct abnormalities and cystic malformations in the male fetus. When referring to these midline cystic malformations of the prostate, previous studies have mostly used the terms MDC and prostatic utricular cysts (PUC) interchangeably. Nevertheless, histologic evaluations of these lesions have suggested that MDCs and PUCs may be distinct pathologic entities.^9^, ^48^ These controversial views reached a peak when Kato et al.^49^ examined five adult cases with midline prostatic cystic lesions immunohistochemically and suggested that there is no evidence to assure that MDCs are actually remnants of the Müllerian ducts and even demonstrated that these cystic lesions probably originate from the prostatic utricle itself. However, pathologic evaluations of excised midline cysts have been inconsistent with some demonstrating a cubo‐columnar epithelial lining comparable to that of the Müllerian ducts,^9^, ^48^ while others reporting a low cuboid to stratified epithelium identical to that of the prostatic utricle.^49^ It is noteworthy that most studies concur on the triple embryologic origin (i.e., urogenital sinus, Wolffian duct, and Müllerian duct) of the prostatic utricle.^50^, ^51^ These multiple embryologic origins can justify the variation in histopathologic findings of cystic lesions in different studies.

MDC is a relatively rare congenital anomaly with a reported 1%–5% prevalence in male autopsies.^52^, ^53^ It usually has no open communication with the posterior urethra and is only attached to the prostatic utricle through a fibrous cord‐like structure.^49^, ^52^ The anatomy of the verumontanum is normal in this condition.^49^ The clinical presentation is varied, including obstructive and irritative lower urinary tract symptoms, UTI, constipation, abdominal pain, hematuria, and ejaculatory problems.^54^, ^55^ Although patients with MDC usually become symptomatic in the third or fourth decades of life, pediatric patients may also present with a wide variety of MDC‐related symptoms (e.g., urinary retention, epididymitis) and even suffer from malignant transformation of these cysts.^9^, ^31^, ^53^, ^56^

Diagnosis of MDC can be challenging in infants. Many differential diagnoses must be considered and ruled out in a boy with a midline cystic lesion, including bladder diverticulum, ureterocele, seminal vesicle or ejaculatory duct cyst, prostatic cyst or abscess, rectal duplication cyst, retroperitoneal tumor, and hydatid cyst.^54^ Abdominopelvic US is usually the initial step of diagnostic evaluation. VCUG and retrograde urethrography are other valuable diagnostic modalities. However, transrectal US and pelvic MRI with an endorectal coil are the best imaging modalities for periprostatic and midline pelvic cysts.^57^

While small asymptomatic cysts are mostly managed conservatively, surgical intervention is warranted in symptomatic patients. Several management strategies have been described in the literature. Open surgery using different approaches (i.e., extravesical or intravesical, transperitoneal, perineal, transrectal, and pararectal) has been performed by several surgeons. Laparoscopic or robotic excision of MDC has also been utilized.^9^, ^58^ Moreover, endoscopic approaches have been utilized in the forms of transurethral aspiration, cold knife incision, fulguration, and dilatation of the utricle.^55^, ^59^, ^60^ There is also a report of trans‐perineal aspiration and sclerotherapy under the guidance of US with successful results.^61^

### 3.3 Ureteral stump syndrome

Ureteral stump syndrome is considered to be a rare pathology with an incidence of only 0.8%–1%. Of these, 1.1%–10% ultimately need intervention.^62^ While some urologists favor complete removal of the refluxing/obstructing ureters,^63^ others argue that partial ureterectomy may be a more suitable choice owing to less complicated surgical method and comparable complications rate.^7^ Nevertheless, the remaining ureteral stump in partial ureterectomy can cause complications. The most common complications related to the ureteral stump are UTI, empyema with or without calculi, pain, hematuria, malignant transformation, and fungal peritonitis.^6^, ^7^, ^63^, ^64^, ^65^, ^66^, ^67^, ^68^

Different approaches for the management of symptomatic ureteral stumps have been introduced. While open and laparoscopic surgeries for stump removal are widely practiced,^6^, ^64^, ^65^, ^69^ some authors have also reported less invasive approaches. Endoscopic sub‐ureteric injection of bulking agents, percutaneous irrigation and drainage with ureteral orifice closure, transurethral fulguration and a combination of endoscopic mucosal fulguration and bulking agent injection are the less invasive options.^64^, ^66^, ^70^, ^71^, ^72^

## 4 CONCLUSION

The prevalent causes of pediatric urinary retention differ from those of the adults. A wide variety of etiologies, ranging from benign behavioral and simple congenital causes to utterly complex anatomical abnormalities or even malignant transformations, can lead to pediatric urinary retention. Considering the complexity of many of these etiologies, their management should be individualized.

## AUTHOR CONTRIBUTIONS

**Pooya Hekmati:** Conceptualization; supervision; writing – original draft. **Hamid Arshadi:** Investigation; resources; writing – review and editing. **Hooman Kamran:** Writing – original draft. **Abdol‐Mohammad Kajbafzadeh:** Investigation; resources; writing – review and editing. **Mazyar Zahir:** Writing – original draft; writing – review and editing. **Hossein Amirzargar:** Investigation; resources; writing – review and editing. **Seyyed Mohammad Ghahestani:** Investigation; resources; writing – review and editing.

## FUNDING INFORMATION

None.

## CONFLICT OF INTEREST STATEMENT

The authors declare that they have no competing interests.

## ETHICS STATEMENT

Ethical approval is not required for case reports in our country according to local guidelines.

## CONSENT

Written informed consent was obtained from the patient to publish this report in accordance with the journal's patient consent policy.

## Data Availability

Not applicable.
